# Impact of a Restriction in Reimbursement on Proton Pump Inhibitors in Patients with an Increased Risk of Gastric Complications

**DOI:** 10.3389/fpubh.2018.00051

**Published:** 2018-02-27

**Authors:** Linda E. Flinterman, Karin Hek, Joke C. Korevaar, Liset van Dijk

**Affiliations:** ^1^NIVEL Netherlands Institute for Health Services Research, Utrecht, Netherlands

**Keywords:** reimbursement, gastric complications, proton pump inhibitors, non-steroidal anti-inflammatory drugs, low-dose aspirin

## Abstract

Governments have several options to reduce the increasing costs of health care, including restrictions for the reimbursement of medicines. Next to the intended effect of reduced costs for medicines, reimbursement restriction can have unintended effects such as patients refraining from their treatment which may lead to health problems and increased use of health care. An example of a reimbursement restriction is the one for proton pump inhibitors (PPIs) that became effective in the Netherlands in January 2012. A major unintended effect of this measure could be that high-risk patients who start with non-steroidal anti-inflammatory drugs (NSAIDs) or low-dose aspirin (aspirin) and who have an increased risk of gastric complications for which they are prescribed PPIs refrain from this PPI treatment. The aim of this study was to evaluate the effect of the reimbursement restriction among high-risk users of NSAIDs or aspirin. Do these patients refrain from their PPI treatment and if so do they have an increased risk of gastric complications? Part of the patients starting with NSAIDs or aspirin have an increased risk of gastric complications due to their age, comorbidities, or co-medication. The incidence of PPI use during the 2 years before the reimbursement restriction (2010 and 2011) and 2 years after the introduction of the reimbursement restriction was compared for patients on NSAIDs or aspirin with an increased risk of developing gastric complications. Impact of age, sex, and social economic status (SES) was taken into account. Hospital admissions due to gastric complications were studied over the same period (2010–2013). Data were obtained from a large population-based primary care database and a hospital database. The use of PPIs in patients with an increased risk of gastric complications who started NSAID/aspirin increased from 40% in 2010 to 55% in 2013. No impact was found of age, sex, or SES. There was no increase in hospital admissions due to gastric complications after the reimbursement restriction. The reimbursement restriction on PPIs was not associated with any detectable unintended effects for patients with an increased risk of gastric complications.

## Introduction

Governments have several policy options to curb the expenses for health care. One of these options is through reimbursement restrictions for medication. After launching a reimbursement restriction for a specific drug, the medication is no longer (fully) reimbursed and patients have to (partly or temporarily) pay for it by themselves. As such, reimbursement restrictions intend to reduce the costs of health care. However, next to the intended reduction of costs for medication, unintended effects can also occur, which need to be studied. Several studies have previously shown the importance of monitoring the effects of reimbursement restrictions on medication (1–5). These restrictions may not always be successful if patients shift to other (more costly) treatments or if they do not lead to clinical benefits (6).

Since January 2012, a reimbursement restriction for proton pump inhibitors (PPI) became effective in the Netherlands (7). Since that date, all patients who start using PPIs have to pay for the first prescription of 14 days themselves. For patients who use PPIs for a period shorter than 6 months this also holds for subsequent PPI prescriptions. Patients who have to use PPIs chronically (>6 months) only have to pay the first prescription, subsequent prescriptions are reimbursed. Before the change in reimbursement, PPIs were reimbursed for all patients. As PPIs are the most frequently prescribed medication, this reimbursement restriction affects two million people in the Netherlands on a yearly basis. This reimbursement restriction fits in a wider range of measurements taken by the Dutch government and general practitioners to reduce costs and unnecessary use of medication, such as more detailed guidelines for PPI prescribing and encouragement of prescribing of generics (8).

Discussions about the possible negative effects of this reimbursement restriction between health care providers and the political arena emerged from the day the restriction was announced in 2011. Opponents were afraid that the reimbursement restriction on PPIs would lead to refrainment from the PPI treatment. Among patients who take PPIs to prevent gastric complications [e.g., patients using non-steroidal anti-inflammatory drugs (NSAIDs) or aspirin] such refrainment may lead to an increase in the risk of gastric complications (9). This increase in gastric complications is not in the best interest of the patients and, at a societal level, might induce costs exceeding the gains of the reimbursement restriction if more patients are hospitalized due to gastric bleeds. Despite these objections, the reimbursement restriction was implemented. Immediately after the start of the reimbursement restriction, two studies showed a decrease in the use of PPIs among patients using NSAIDs or aspirin with an increased risk of gastric complications in the first half of 2012 and estimated an increase in gastric complications based on data from other countries (10, 11). However, longer-term trends and Dutch data on hospital admissions for gastric complications were lacking.

The aim of this study was to evaluate the effect of this reimbursement restriction for PPIs for patients with an increased risk of gastric complications due to the use of NSAID/aspirin during 2 years before and after the reimbursement restriction.

## Materials and Methods

### Data

Data from the NIVEL Primary Care Database were used (NIVEL-PCD). NIVEL-PCD collects longitudinal data from patients of general practices in the Netherlands since 2001 (12). The number of general practices participating in NIVEL-PCD increased from 120 in 2001 to 485 in 2014. The number of patients for whom information is available increased from 500,000 to over 1.9 million between 2001 and 2014. Patients and their medication use can be followed over time. We used the following data: prescription data, diagnoses, age, sex, and social economic status (SES). The latter is based on neighborhood level (13). The prescription data consist of all prescriptions made by the general practitioner, coded according to the Anatomical Therapeutic Chemical (ATC) Classification Index and the prescription date (14). Diagnoses are coded according to the International Classification of Primary Care-codes (15).

### Study Population

We selected general practices that delivered data for at least three subsequent years within the period 2009–2014. We needed 3 years per general practice to be able to construct four cohorts, one for each year in the period 2010–2013. Per cohort we selected all patients aged 20 and older those were registered in the year of the cohort as well as in the year before and 6 months after the cohort. For example, for the 2011-cohort, we selected patients who were registered in 2010 (year before cohort), 2011 (year of the cohort), and at least the first 6 months of 2012 (period after cohort). We included data from the year before the cohort-year to determine whether patients were new users of NSAID/aspirin with an increased risk of gastric complications (from here “high-risk patients”) in the cohort-year, i.e., they did not receive any prescription in the pre-cohort-year for NSAID or aspirin. Data from at least 6 months after the cohort-year were used to determine whether PPIs were used for more than 6 months, as after 6 months a PPI user becomes a chronic user and they receive reimbursement after the first prescription. So, we could determine whether a patient became a chronic user even for patients who started with a PPI in the last months of a cohort-year.

We selected patients with at least one prescription of NSAID or aspirin (ATC-codes: M01A—except M01AC06, M01AC56, M01AH01, M01AH02, and M01AX01—B01AC06, B01AC08, B01AC30, N02BA01, N02BA15, N02BA51, N02BA65) and who were at risk of gastric complications according to the guideline “Gastrointestinal complaints” of the Dutch College of General Practitioners (Table 1) (16).

**Table 1:** Definition of patients with an increased risk of gastric complications according to the Dutch guideline “Gastric complications.”

Non-steroidal anti-inflammatory drugs users	Prescribe proton pump inhibitor (PPI) if:
	Patient has a history of ulcers gastric complications
	The patient is 70 years of older
	If the patient has two or more of the following risk factors:
	– 60 to 70 years old
	– Has disabling rheumatoid arthritis,^a^ heart failure or diabetes
	– Uses one of the following medications: coumarins, clopidogrel, prasugrel, acetylsalicylic acid (as platelet inhibitor), systemic glucocorticoids, SSRIs, venlafacin, duloxetine, trazodone, or spironolactone.
**Low-dose aspirin users**	**Prescribe PPI if:**
	The patient is 80 years or older
	The patient is 70 years or older and uses coumarins, clopidogrel, prasugrel, ticagrelor, systemic glucocorticoids, SSRIs, venlafaxine, duloxetine, trazodone, or spironolactone
	The patient is 60 years or older and has a history of ulcers or gastric complications

*^a^Disabling rheumatoid arthritis was defined according to Nielen et al. (17)*.

### New and Current Users

We defined two types of high-risk users of NSAID and/or aspirin: new users and current users. New users were those who started with the use of NSAID/aspirin in the year the patient entered the cohort. Current users were those who already used PPIs and/or NSAID/aspirin in the year before the patient entered the cohort. The percentage of PPI-users among high-risk patients newly or currently using NSAIDs or aspirin was calculated per 3 months for the period 2010–2013. Percentages of PPI use were also calculated for subgroups of age, sex, and SES. Trends over the years were analyzed with a Chi^2^ test overall and for the different subgroups.

### Hospitalizations

In the Netherlands, hospital care is registered in the form of diagnosis treatment combinations (DBCs). A DBC contains the total of treatments from the first consultation in the hospital for a certain condition up to the last check. These DBCs are registered in the DBC information system (DIS). All hospitals in the Netherlands are obliged to deliver their data on the usage of care expressed in DBCs and provided with the starting date, to the DIS register. We used data over the period 2010–2013 to estimate the number of hospital admissions for gastric complications. We compared the numbers of such hospitalizations before and after the reimbursement restriction and related it to the amount of PPIs prescribed in general practice.

### Sensitivity Analysis Prescriptions

The NIVEL Primary Care Database contains prescription data as prescribed by the general practitioner. Information on the question whether or not patients filled their prescription at the pharmacy is lacking. It is possible that fewer patients collected their PPI medication after the reimbursement restriction. We assumed that patients with only one prescription and no second prescription either only have one prescription or did not collect their medication from the pharmacy and therefore did not fulfill their second prescription. If high-risk patients refrain from using PPIs due to the reimbursement restriction, there should be more patients with only one prescription when the reimbursement restriction became effective. To check this, we performed a sensitivity analysis where we compared the number of patients with only one prescription of PPIs before and after the reimbursement measure.

### Sensitivity Analysis Gastric Complications

To determine the number of hospitalizations due to gastric complications, we calculated the number of hospitalizations per 10,000 inhabitants of the Netherlands per year for the period 2010–2013. Because not all data from the hospitals were delivered already to the DIS registry for the years 2012 and 2013, they received approximately 60–85% of the data; we recalculated the numbers received from 2012 and 2013 to 100%. To adjust for some possible selection in the delivery of the data, we added and deducted 10% from the number of complications per 10,000 inhabitants. We performed these analyses for the total population as well as for the subgroups of age, sex, and SES.

## Results

### Patients

Of all patients of 20 years and older registered in the NIVEL-PCD, 15% had a prescription of NSAID or aspirin. In all four annual cohorts about 30% of these were high-risk patients according to the NHG guidelines. In the four cohorts for 2010–2013 up to 16,013 patients were included (Table 2). The number of patients per cohort is steadily rising due to the increased number of participants of NIVEL-PCD.

**Table 2:** Percentage of users of proton pump inhibitors (PPIs) among users of non-steroidal anti-inflammatory drugs or aspirin with an increased risk of gastric complications for several subgroups.

	Year				
	2010	2011	2012	2013	Chi^2a^
*N* total	5,545	12,906	14,863	16,013	
% Using PPIs	65	68	69	74	<0.01
**Sex**				
Men	65%	66%	66%	71%	<0.01
Women	65%	69%	71%	76%	<0.01
**Age**				
60–69 years	74%	78%	76%	77%	0.14
70–79 years	76%	77%	79%	82%	<0.01
≥80 years	46%	53%	58%	67%	0.01
**Social economic status**				
Very high	64%	68%	71%	73%	<0.01
High	65%	68%	67%	73%	<0.01
Average	67%	67%	72%	77%	<0.01
Low	63%	67%	68%	71%	<0.01
Very low	66%	68%	70%	74%	<0.01

*^a^Chi^2^ for trend over time*.

### Current Users of NSAID/Aspirin

**Figure 1:**
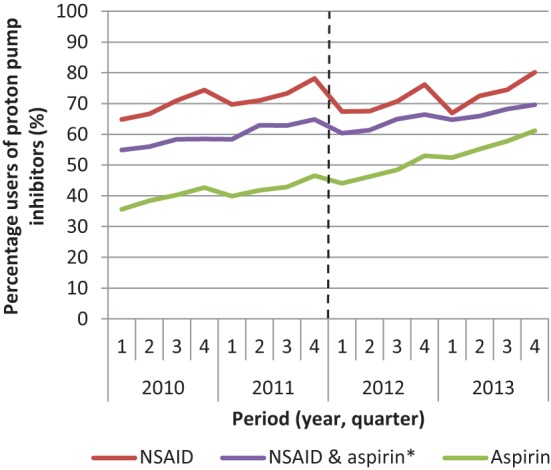
Percentage of patients using proton pump inhibitors among patients with an increased risk of gastric complications that use non-steroidal anti-inflammatory drugs (NSAID) or aspirin or both in the period 2010–2013. *Users of NSAID and aspirin use both medications at the same time.

In 2010, 65% of the high-risk patients used a PPI; in 2013, this percentage increased to 74% (Table 2). This increase was stronger for aspirin users (from 36 to 61%) compared to NSAID users (from 65 to 80%) (Figure 1). A temporary decrease in the percentage of users is seen in every first quarter of every year. Yet, the overall trend of PPI use shows a clear increase.

### New Users of NSAIDs/Aspirin

**Figure 2:**
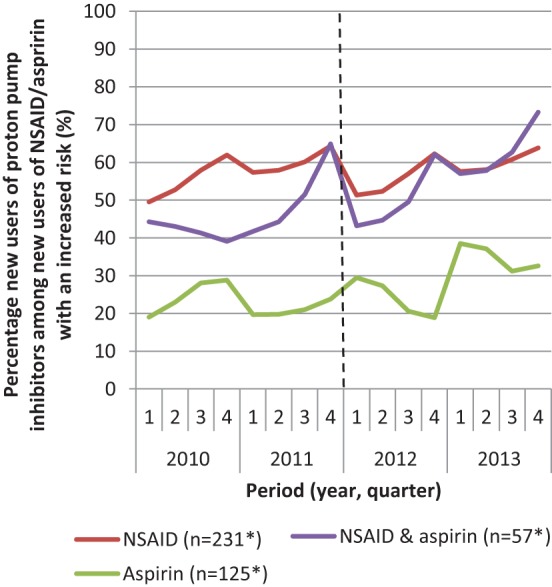
Percentage of new users proton pump inhibitors among patients with an increased risk of complications that start with non-steroidal anti-inflammatory drugs (NSAID) or aspirin or both in the period 2010–2013. *Mean number of patients per quarter.

The reimbursement restriction only has an effect on those who are new high-risk patients who have to start using PPIs, as these patients have to pay for at least their first prescription. Figure 2 shows the percentage of new high-risk patients who filled a prescription of PPIs over time. This figure shows an increase in the number of new PPI-users from 40 to 55% among new high-risk patients. These patients conjointly start using PPIs and NSAIDs/aspirin.

### Current Users of NSAID/Aspirin and New Users of PPIs

Of the patients who use NSAID/aspirin when they enter the cohort but who were not using a PPI at the moment they enter the cohort, 10% starts using PPIs over time. There was no change in this percentage in the different cohorts. Hence, if patients at start of NSAID/aspirin chose to not use PPIs, 90% did not start the use of PPIs at a later moment in time.

### Sensitivity Analysis of PPI Use

Over time among the patients who start using PPIs, the percentage of patients with only one prescription of PPIs remained stable at 25%. This suggests that the same proportion of patients decided to refrain from using a PPI (after only one prescription) before and after the reimbursement restriction.

### PPI Use among Subgroups of High-Risk Patients

For all studied subgroups of age, sex, and SES, the percentage of PPI-users remained stable or increased over time (Table 2). This increase was significant in all groups except for those between 60 and 69 years of age. This group of patients already had the highest percentage of PPI use. Therefore, there might have been a ceiling effect as it is unlikely that 100% of the high-risk patients will use PPIs.

### Number of Hospitalizations for Gastric Complications

The number of hospitalizations for gastric complications decreased in the period 2010–2013 (Table 3). This decrease was still present after correction for possible missing data (sensitivity analysis). This decrease was seen in all subgroups: men, women, different age groups, and for different levels of SES.

**Table 3:** Number of gastric complications for which a hospital admission was required per year.

	Year			
	2010	2011	2012	2013
	*N* per 10,000 (95% CI)	*N* per 10,000 (95% CI)	*N* per 10,000 (95% CI)	*N* per 10,000 (95% CI)
Total	4.6 (4.5–4.7)	4.5 (4.4–4.6)	3.7 (3.6–3.8)	3.2 (3.1–3.3)
Range −10%^a^			4.4 (4.3–4.5)	4.2 (4.1–4.3)
Range +10%^a^			3.1 (3.0–3.2)	2.6 (2.5–2.7)
**Age**			
20–59	3.8 (3.7–3.9)	4.0 (3.9–4.1)	2.6 (2.5–2.7)	2.1 (2.0–2.2)
60–69	5.3 (4.9–5.6)	4.8 (4.5–5.1)	4.6 (4.3–4.9)	4.4 (4.1–4.7)
70–79	7.2 (6.7–7.7)	6.4 (6.0–6.9)	7.2 (6.7–7.7)	6.4 (5.9–6.8)
80+	9.3 (8.6–10)	7.3 (6.6–7.9)	8.6 (7.9–9.3)	8.0 (7.3–8.6)
**Social economic status**				
Very high	3.6 (3.3–3.8)	3.7 (3.5–4.0)	2.9 (2.7–3.1)	2.5 (2.3–2.7)
High	3.7 (3.5–4.0)	3.8 (3.5–4.0)	3.0 (2.7–3.2)	2.9 (2.7–3.1)
Average	4.4 (4.1–4.7)	4.1 (3.8–4.4)	3.3 (3.1.3.6)	3.2 (3.0–3.4)
Low	4.6 (4.3–4.8)	4.4 (4.2–4.7)	3.4 (3.1–3.6)	3.3 (3.0–3.5)
Very low	5.9 (5.6–6.2)	5.8 (5.6–6.1)	5.1 (4.8–5.3)	3.8 (3.6–4.0)

*^a^As a sensitivity analysis incidence rates were calculated assuming the registered number of hospital admissions was 10% to high or low*.

## Discussion

The restriction in the reimbursement of PPIs in the Netherlands did not lead to a reduced use of PPIs in patients who are at risk of gastrointestinal complications because of NSAID and/or aspirin use. On the contrary, an increase in the use of PPIs among high-risk patients was seen in the period 2010–2013 for the total population of users as well as for subgroups of patients based on age, sex, and SES. In the same period, the number of hospitalizations for gastric complications decreased.

Since 2009, there has been increased attention in the Netherlands for the necessity to prescribe PPIs for high-risk patients. In that year, a report on medication safety was published (HARM Wrestling report) and based on this report, renewed national GP guidelines for treatment of high-risk patients were published (16, 18). The attention the report got in the medical world and in the media, most likely, had a positive effect on the use of PPIs in the high-risk population. While the reimbursement restriction might have had a negative effect of the use of PPIs, by the time, the reimbursement restriction was introduced in 2012, these guidelines were probably well known. Moreover, in the period before and just after the start of the reimbursement restriction, there was a lot of publicity on the potential negative effects of refraining from PPIs. This might have led to an increased awareness among prescribers and patients on the importance of PPIs and subsequently to an increase in use. Unfortunately we are not able to distinguish between the effects of the extra publicity, changes in guidelines and the reimbursement restriction. Still, the overall effect was an increase in use of PPIs. Other previous reimbursement changes concerning PPIs in the Netherlands resulted in an increase in the use of PPIs as well (5, 8). However, these measures did not increase the financial burden of patients.

In this study, we saw a temporary decrease in percentage in every first quarter of every year. Previous reports showed a similar decrease in the use of PPIs in the first 6 months after the introduction of the restriction (10, 11). These studies concluded that the reimbursement restriction led to a reduced use of PPIs. However, the timeframe of these studies was too limited. They analyzed the period between 6 months before until 6 months after the introduction of the reimbursement restriction, and thus missed seasonal variation. The annual decrease in the use of PPIs we observed at the start of each new calendar year might be caused by the fact that patients fill their prescriptions at the end of the calendar year, because they used their deductible excess for that year, while as of January 1 of the next year, the deductible excess for that year will start and patients have to pay the health care costs themselves. Our results argue for a sufficiently long period of follow-up in evaluations of reimbursement measures before and after the introduction of a measure.

In the Netherlands, it is possible to buy PPIs over the counter at pharmacies. Information about the sale of these PPIs was lacking in this study. However, the aim of this study was to see whether high-risk users of NSAIDs or aspirin refrain from their PPI prescription because of the reimbursement restriction. If we would have seen a decrease in PPI prescriptions, this might have been compensated by over the counter sales. However, we observed an increase in the number of PPI prescriptions in this group of patients.

NIVEL-PCD contains data from up to 485 general practices in the Netherlands, which is a representative sample of 10% of the Dutch population according to age and sex. Because of this large pool of systematically collected data, we are able to show trends in time. Thereby, NIVEL-PCD also contains information about the medical history of the patient. However, NIVEL-PCD only contains prescriptions from the general practitioner and only a limited amount of prescriptions from specialists. Therefore, prescriptions of PPIs could be missing in our data. However, it is not likely that the ratio between the number of prescriptions for PPIs from general practitioners and specialists changed over time. Therefore, the fact that we did not have all information on prescriptions from medical specialists cannot explain the increase in use of PPIs in this study.

Social economic status was determined by the first four numbers of the postal code of the patient’s address (13). As such, SES was determined for the district patients lived in and not for the individual patient.

For this study, we chose to report the percentage of patients with a prescription for PPIs instead of DDDs used over time. The influence of this reimbursement restriction was that patients might choose not to start using PPIs due to the costs. Starting on a lower dose hardly affects the amount patients have to pay as the main part of the costs are the handling costs for the pharmacy and not the costs of the medication itself. It is therefore unlikely that general practitioners would prescribe lower doses because of this reimbursement restriction. Therefore, reporting the percentage of patients using PPIs was justified in this case.

The number of hospitalizations due to gastric complications was based on all hospital records in the Netherlands. We could not determine which hospitalizations were for our target population, i.e., high-risk patients who did not use PPIs, as the DBC’s imply the reason for hospitalization (gastric bleed) but not the cause of it. To our knowledge, there were no other explanations for a decrease in hospitalizations for gastric bleeds in the years 2010–2013. As the results from the prescriptions and hospital records pointed in the same direction and there were no clear other causes of a decrease in the number of gastric complications, the correlation between these two separate results seems valid.

The question arises whether a similar reimbursement restriction can be used on other medications as well? This might be possible under certain conditions. Two conditions why this reimbursement restriction seemed to be effective come to mind. First, most patients only had to pay the first period of use. Those who used PPIs for more than 6 months only have to pay for the first 2 weeks, which is a maximum of 14 euros. This is a fairly small amount of money knowing that patients in the Netherlands already have to pay a deductible excess of 385 euros. Next, there are more medications such as benzodiazepines that patients have to pay out of pocket, which patient do when they need the medication (2). So patients are used to pay some of the costs themselves. Second, the reimbursement restriction on PPIs came shortly after the publication of the HARM Wrestling report. This made general practitioners more aware of the need to prescribe PPIs to these high-risk patients.

Our findings are in line with two reviews that showed that reimbursement restrictions could be successful when: (1) they are based on research quantifying the possible harm and benefits and (2) the measure is not too costly for individual patients (1, 19, 20). Therefore, for possible future reimbursement restrictions it is recommended to consider the extra financial burden for individual patients and the possible negative effects of the restrictions before implementing the restriction. Additionally, it is recommended to monitor the longer-term effects after implementation to see the real effects of the restriction (1, 19, 20).

## Conclusion

This study showed that—under certain preconditions—reimbursement restrictions can be implemented without unintended negative effects. The reimbursement restriction in this study did not lead to a lower use of PPIs in high-risk patients and no increase in hospitalization due to gastric complications was seen. Instead, the number of patients starting with PPIs increased. Our results show that monitoring reimbursement restrictions with a sufficient follow-up period is important to evaluate the intended and possibly unintended effects.

## Ethics Statement

The study was carried out according to Dutch legislation on privacy. The privacy regulation of the NIVEL-PCD was approved by the Dutch Data Protection Authority. According to Dutch legislation, obtaining informed consent and/or approval by medical ethics committee is not obligatory for observational studies. This study has been approved by the applicable governance bodies of NIVEL-PCD under number NZR-00314.039.

## Author Contributions

LF performed the statistical analysis and drafted the manuscript. KH assisted with statistical analysis and drafted the manuscript. JK and LD participated in the design and coordination of the study. All authors read and approved the final manuscript.

## Conflict of Interest Statement

The authors declare that the research was conducted in the absence of any commercial or financial relationships that could be construed as a potential conflict of interest.
